# The Role of Interventional Radiology in the Management of Late Postpancreaticoduodenectomy Hemorrhage

**DOI:** 10.1155/2020/8851950

**Published:** 2020-12-14

**Authors:** Liang Zhang, Jun Wang, Jinhua Jiang, Jialin Shen

**Affiliations:** Department of Interventional Oncology, Renji Hospital, School of Medicine, Shanghai Jiao Tong University, No. 160 Pujian Road, Pudong, Shanghai 200127, China

## Abstract

**Objective:**

To explore the role of interventional radiology (IR) in the management of late postpancreaticoduodenectomy hemorrhage (PPH).

**Materials and Methods:**

Patients who had late PPH (occurring >24 h after index operation) managed by the IR procedure in our institution between 2013 and 2018 were retrospectively analyzed.

**Result:**

Hired patients who were diagnosed with grade B (*n* = 10) and C (*n* = 22) late PPH underwent 40 transcatheter arterial angiographies (TAA). The overall positive rate of angiography was 45.0% (18/40). Eighteen transcatheter arterial embolizations (TAEs) were performed, and the technical success rate was 88.89% (16/18). The rebleeding rate after embolization was 18.8% (3/16), and no severe procedure-related complications were recorded. The overall mortality of late PPH was 25.0% (8/32).

**Conclusion:**

Nearly half of hemorrhagic sites in late PPH could be identified by TAA. TAE is an effective and safe method for the hemostasia of late PPH in patients with positive angiography results.

## 1. Introduction

Hemorrhage is a less frequent but potentially fatal complication following pancreaticoduodenectomy (PD). The reported incidence of post-PD hemorrhage (PPH) ranges from 1.5% to 15% [1–4], while it accounts for 10% to 38% of overall mortality [5, 6]. The International Study Group for Pancreatic Surgery (ISGPS) classifies PPH as early or late, according to its onset [7]. Early PPH occurs less than 24 h after PD, and late PPH occurs more than 24 h after the index operation. Early and late PPH should be regarded as two different types of surgical morbidity in PD, because early PPH is most likely caused by inadequate or incomplete hemostasis and can be effectively treated by reoperation [7, 8], while late PPH is typically associated with postoperative complications such as intra-abdominal abscess, erosion of a peripancreatic vessel secondary to a pancreaticobiliary fistula, ulceration at the site of an anastomosis, or development of an arterial pseudoaneurysm [9, 10]. The mortality rate of late PPH is nearly 50%, and it is among the most devastating of the post-PD morbidities [11, 12].

In recent years, an interventional radiology (IR) approach has been considered a preferred treatment for late PPH, with the advantages of being minimally invasive and highly effective and low complication rate [13–15]. However, given the low incidence of late PPH, the data assessing the role of IR in the management of late PPH are still limited. Thus, the aim of this study was to evaluate the diagnostic value of transcatheter arterial angiographies (TAA) as well as the efficacy and safety of transcatheter arterial embolizations (TAEs) for late PPH.

## 2. Materials and Methods

The study protocol was approved by the ethics committee of the local institution. Data from all patients who had late PPH and underwent IR treatment between January 2013 and December 2018 at our institution were retrospectively reviewed. Late PPH was defined based on the definition of PPH proposed by the ISGPS [7]. Diagnostic procedures for late PPH included routine blood tests, drain-fluid cultures, abdominal ultrasound, contrast-enhanced computed tomography (CT), upper GI endoscopy, and angiography. CT angiography was performed in all the patients before IR treatment, to evaluate the site of bleeding. All the cases were classified into ISGPS grades A, B, and C and categorized into intraluminal, extraluminal, or both intra- and extraluminal hemorrhage. Intraluminal hemorrhage was defined as bleeding inside the gastrointestinal (GI) tract or the presence of hematemesis or melena; extraluminal hemorrhage was defined as intra-abdominal bleeding outside the GI tract.

Angiography and endovascular treatment were performed using a Siemens Artis Zeego system (Siemens Healthineers, Forchheim, Germany) as follows: First, all patients underwent selective and superselective abdominal visceral angiography, including the portal venous phase. Then, the angiography was reviewed by two experienced radiologists to determine the location of the bleeding and the potential for TAE on consensus. If the bleeding site was not identified or the bleeding was not amenable to TAE, conservative therapy, endoscopy, reangiography, or relaparotomy was applied according to the hemodynamic stability of the patient and the location of the bleed. TAE was performed in a superselective catheter position by coil embolization (fibered platinum coils and interlock detachable coils; Boston Scientific, Cork, Ireland) and/or gelatin sponge (Jinling Pharmaceutical Co. Ltd., Nanjing, China). No covered vascular stent was used in the study population. The coil embolization techniques included distal and proximal embolization of vascular irregularities or bleeding sites, embolization of vascular stumps created during surgery, and direct coil embolization of a pseudoaneurysm. Coils were placed until complete exclusion of the bleeding site was obtained, as defined by the disappearance of the vascular abnormality on repeat angiography. Gelatin sponge embolization was most often applied for lower gastrointestinal hemorrhage originating from the branches of the superior mesenteric artery or for coil-assisted embolization. For hemorrhage recurring after the IR procedures, repeat IR or relaparotomy was performed according to the severity of the bleed and the condition of the patient. Technical success was defined as effective embolization in the hemorrhagic artery, with no direct or indirect signs of hemorrhage on angiography immediately following the intervention.

Clinical variables associated with hemorrhage and postoperative management were noted from the medical history. Operative variables related to hemorrhage were extracted from the anesthesia record and operative reports. Pathology reports were reviewed to determine the final pathology diagnosis. The findings and results of the IR procedures were reviewed from the picture archiving and communication system and the procedure records. The patients' clinical conditions and laboratory test results were documented until discharge or death as well as during the appointments at the outpatient clinic up to the recorded period. Complications and mortality that occurred during the course of the study were documented.

Statistical analysis was performed using SPSS version 13.0 (SPSS Inc., Chicago, IL, USA). All quantitative variables are expressed as means ± standard deviations, and categorical variables are expressed as frequencies and percentages. The positive rates of angiography and the mortality rates according to the bleeding location were compared by using the Fisher exact test. *p* values < 0.05 were considered significant.

## 3. Results

A total of 32 patients with late PPH managed by an IR procedure in our institution from January 2013 to December 2018 were included. Their characteristics and the factors related to PPH are presented in Table 1.

Ten patients were diagnosed with grade B PPH and IR treatment was performed immediately after failing in conservative therapy, and 22 patients were diagnosed with grade C PPH and IR treatment was performed as soon as the diagnosis was established.

**Figure 1:**
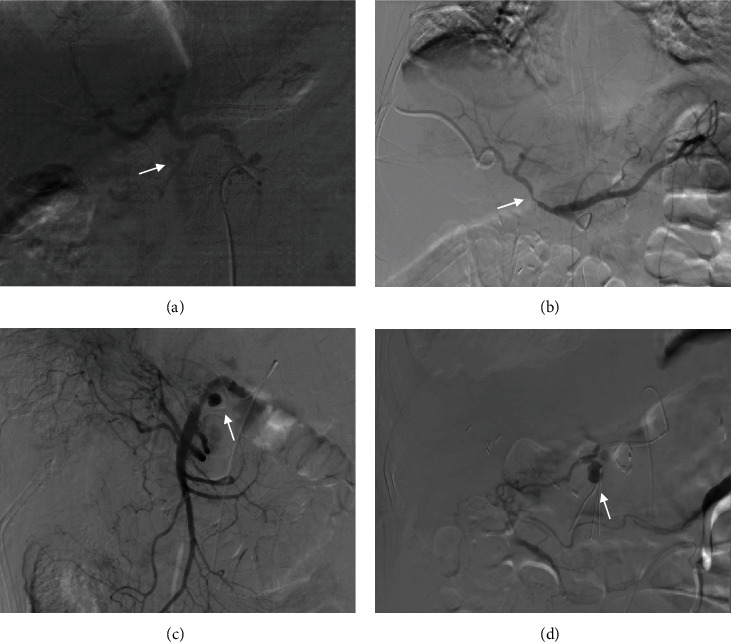
Angiographic manifestation of late postpancreaticoduodenectomy hemorrhage: (a) angiography in a 63-year-old man with a mixed intra- and extraluminal hemorrhage shows extravasation of the contrast medium in the proximal portion of the gastroduodenal artery (GDA) (arrow); (b) angiography in a 58-year-old woman with a mixed intra- and extraluminal hemorrhage shows focal stenosis of the common hepatic artery (arrow); (c) angiogram in a 52-year-old man with extraluminal hemorrhage shows a pseudoaneurysm in the superior mesenteric artery and irregularity of the artery wall (arrow); (d) angiography in a 61-year-old woman with intraluminal hemorrhage shows a narrow-neck pseudoaneurysm in the GDA (arrow).

In total, 40 angiographies (including 8 reangiographies) were performed in the 32 study patients. Eighteen hemorrhagic foci in 15 patients were detected by TAA. The overall positive rate of angiography was 45% (18/40). The positive rate of TAA for intraluminal hemorrhage, extraluminal hemorrhage, and both intra- and extraluminal hemorrhage was 25% (3/12), 53.3% (8/15), and 53.8% (7/13), respectively (Table 2). No significant difference was found in the positive rate of angiography among different hemorrhage locations (*p* = 0.250). Angiographic positive findings for PPH included extravasation of the contrast medium (7/18), pseudoaneurysm (8/18), and focal stenosis of an artery or irregularity of arterial walls (5/18) (Figure 1). PPH originated from the gastroduodenal artery in 7 cases, the common hepatic artery in 3, the proper hepatic artery in 2, the superior mesenteric artery in 2, the splenic artery in 2, and the right hepatic artery in 2. Eight patients underwent repeat angiography, and the interval between the studies ranged from 12 to 72 h. Two of these patients had negative findings in both angiographies, 2 had positive findings in both angiographies, 3 showed negative results the first time and positive results the second time, and 1 appeared positive the first time but negative the second time.

The 17 patients whose angiographic results were negative underwent endoscopy examination or relaparotomy, and 14 hemorrhagic sites were detected. The bleeding sources were detected at the pancreatic cut surface or the anastomotic stoma of the pancreaticojejunostomy in 8 cases, at the gastric or duodenal ulcer in 3 cases, at the tributaries of the portal vein in 2 cases, and at varices of the esophageal and gastric fundus in 1 case. The bleeding source in 3 patients was not confirmed.

**Figure 2:**
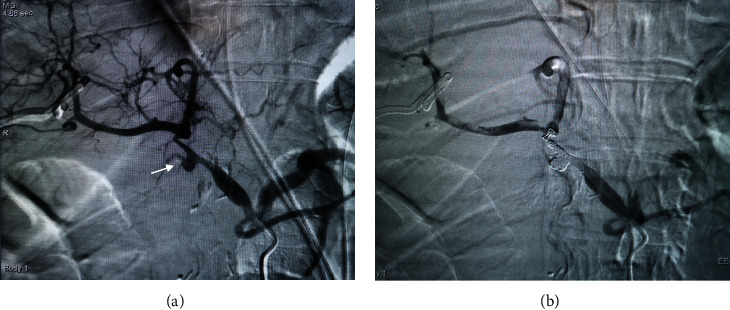
Endovascular treatment of late postpancreaticoduodenectomy hemorrhage. A 64-year-old man with hemorrhage through surgical drain 30 days after surgery: (a) selective celiac axis arteriography revealed a pseudoaneurysm that occurred in the stump of the gastroduodenal artery (arrow) and (b) reangiography showed that the pseudoaneurysm was occluded by coil embolization. The patient was discharged from the hospital 15 days after endovascular treatment, without suffering from rebleeding and any severe procedure-related complication during 1 year of follow-up.

The 15 patients with positive results on angiography underwent 18 TAEs with a technical success rate of 88.89% (16/18) (Figure 2). Embolization was not effective in 2 cases of attempted coil embolization. One was a mixed hemorrhage in a patient whose common hepatic artery was severely eroded by abdominal infection. It was not possible to place the coil in the proximal bleeding site in this case, and the patient did not survive the PPH. The other patient required relaparotomy after angiography because it was not possible to pass the microcatheter through the aneurysmal neck at the root of the SMA. This patient died of sepsis with multiple organ failure the second day after relaparotomy.

Three patients (23.08%) experienced postembolization rebleeding, and 2 of them died with recurrent hemorrhage. One patient developed a hepatic abscess that was drained percutaneously and recovered in 2 months. No other procedure-related hepatic complications were recorded.

The mean follow-up time was 99 days (range 27 to 612 days). The mortality rate of late PPH was 25.0% (8/32). Mortality rates according to the hemorrhage location are shown in Table 2. There was no significant difference when comparing mortality among different hemorrhage locations (*p* = 0.073). Three patients died with overwhelming abdominal sepsis. Three deaths were directly related to massive uncontrolled hemorrhage from the original site, and 2 patients died of massive postembolization rebleeding.

## 4. Discussion

Although the incidence of post-PD hemorrhage has been declining [13, 16], late PPH remains a severe complication that is responsible for a substantial increase in perioperative mortality [17]. To date, there are no definite guidelines for the treatment of late PPH, but all agree that early recognition and intervention are essential to survival [18–21]. In the present study, the efficacy of IR in the management of late PPH was investigated. Our results showed that nearly half of hemorrhagic sites in late PPH could be identified by TAA. TAE is an effective and safe method for the hemostasia of late PPH in patients with positive angiography results.

Previous studies have shown that the TAA can correctly identify 70–90% of arterial hemorrhages [12, 15]. However, the positive rate of angiography was only 45% in the present study. There are some reasons that could account for this: first, because of the intermittent nature hemorrhage [14], a single diagnostic angiography may yield false-negative results. Three patients in the present study showed negative results on a first angiogram while positive results on a second angiogram. Iswanto and Nussbaum [22] also suggested that if a TAA fails to clarify the hemorrhage site, a repeated angiography can be performed 6–24 h later. Second, not all the bleeding originated from arteries. Three patients in the present study presented hemorrhage from veins (variceal vein and portal vein hemorrhage). Furthermore, TAA can detect signs of a contrast agent overflow only when the velocity of the hemorrhage is more than 0.5 mL/min. For some cases, the hemorrhage may be missed due to the low velocity.

According to previous studies concerning the effect of IR in the treatment of late PPH, the rates of technical success and rebleeding are 82% to 100% and 7% to 30%, respectively, and the reported mortality rate ranges from 7% to 54% [21, 23, 24]. In the present study, the technical success rate of embolization for late PPH was 88.89% (16/18), the rebleeding rate was 23.08% (3/13), and there were no severe hepatic complications recorded. The mortality rate of late PPH was 25.0% (8/32). Thus, IR is a safe and effective means of treatment in late PPH. Roulin et al. [25] have commented on the safety of early angiography and endovascular treatment and recommended it as the procedure of choice, noting that surgery should be the primary treatment only where IR is not available or where patients cannot be stabilized for an interventional treatment. There was no significant difference between IR and laparotomy in terms of complete hemostasis (80% vs. 76%, *p* = 0.350), but a significant decrease in mortality in IR vs. laparotomy (22% vs. 47%, *p* = 0.020).

Management of late PPH should be done according to the site of bleeding. Patients who presented with both intra- and extraluminal hemorrhage showed mortality greater than 50% in the present study. This is presumably because mixed intra- and extraluminal hemorrhage was usually associated with a persistent postoperative pancreaticobiliary fistula or serious abdominal infection, leading to erosion of adjacent vessels and resulting in massive blood loss or recurrent hemorrhage after embolization [12, 14]. In such cases, multiple angiographies may be essential to confirm that all bleeding sources are identified, and more aggressive embolization can be performed. Extraluminal hemorrhage was presented in 43.75% (14/32) of the patients in this study, which is reported as the most common site of late PPH [5]. Extraluminal hemorrhage is frequently caused by vessel erosion due to abdominal infection, pancreatic fistula, or rupture of a pseudoaneurysm. In most of these cases, it was possible to visualize and embolize the bleeding vessels during IR, resulting in a decrease in the mortality rate in extraluminal PPH. Intraluminal PPH is usually associated with ulceration at the anastomotic site or anastomotic fistula, which showed a low detection rate at angiography (25%) in the present study. Thus, upper GI endoscopy is recommended when intraluminal hemorrhage is suspected, if the patient did not show signs of hemodynamic instability [26].

There are some limitations in the study. First of all, this is a retrospective study, and a prospective randomized clinical trial comparing IR and other therapeutic methods in the treatment of late PPH should be planned. Second, the study population was fairly small. Third, because covered vascular stents were not available in our center, only coil and gelatin sponge embolization was performed, and previous studies have shown that placing a stent graft rather than using permanent embolic agents allows the hepatoportal blood flow to be maintained and reduces complications [27, 28].

In summary, IR can be the preferred method for the safe and effective treatment of late PPH. TAA could identify the hemorrhagic sites in nearly half of late PPH cases. For patients with positive angiography results, late PPH can be managed by TAE with acceptable morbidity and mortality.

## Figures and Tables

**Table 1:** Characteristics of patients presenting with late postpancreaticoduodenectomy (PD) hemorrhage.

Variables	*n*
Gender	
Male	25
Female	7
Age (year)	54 ± 12.4 (15-73)
Pathology	
Villus-tubiform adenoma of duodenal papilla	2
Solid pseudopapillary neoplasm of the pancreas	2
Pancreatic ductal adenocarcinoma	16
Pancreatic neuroendocrine tumor	3
Duodenal stromal tumor	3
Pancreatic intraductal papillary mucinous neoplasm	2
Duodenal periampullary adenocarcinoma	2
Inflammatory hyperplasia	1
Pancreatic cystic disease	1
Intraoperative blood loss (mL)	545.24 ± 428.34 (200-2000)
Time of onset after PD (day)	18.22 ± 9.25 (2-40)
Drop of hemoglobin (g/L)	30.14 ± 13.58 (7-62)
Sentinel bleed	8
Location	
Intraluminal	11
Extraluminal	14
Intra- and extraluminal	7
Biliary fistula	2
Pancreatic fistula	4
ISGPS grade	
Grade B	10
Grade C	22

ISGPS: International Study Group for Pancreatic Surgery.

**Table 2:** Angiographic detection and mortality rates according to the hemorrhage location.

Bleeding location	Patients	Number of angiographies	Positive rate	Mortality
Intraluminal	11	12	25.0% (3/12)	1 (9.1%)
Extraluminal	14	15	53.3% (8/15)	3 (21.4%)
Intra- and extraluminal	7	13	53.8% (7/13)	4 (57.1%)
Total	32	40	45.0% (18/40)	8 (25%)

## Data Availability

The data used to support the findings of this study are available from the corresponding author upon request.

## References

[1] Feng J., Chen Y. L., Dong J. H., Chen M. Y., Cai S. W., Huang Z. Q. (2014). Post-pancreaticoduodenectomy hemorrhage: risk factors, managements and outcomes. *Hepatobiliary & Pancreatic Diseases International*.

[2] Mañas-Gómez M. J., Rodríguez-Revuelto R., Balsells-Valls J. (2011). Post-pancreaticoduodenectomy hemorrhage. Incidence, diagnosis, and treatment. *World Journal of Surgery*.

[3] Wellner U. F., Kulemann B., Lapshyn H. (2014). Postpancreatectomy hemorrhage-incidence, treatment, and risk factors in over 1,000 pancreatic resections. *Journal of Gastrointestinal Surgery*.

[4] Tien Y. W., Lee P. H., Yang C. Y., Ho M. C., Chiu Y. F. (2005). Risk factors of massive bleeding related to pancreatic leak after pancreaticoduodenectomy. *Journal of the American College of Surgeons*.

[5] Balachandran P., Sikora S. S., Raghavendra Rao R. V., Kumar A., Saxena R., Kapoor V. K. (2004). Haemorrhagic complications of pancreaticoduodenectomy. *ANZ Journal of Surgery*.

[6] Kapoor V. K. (2016). Complications of pancreato-duodenectomy. *Rozhledy v Chirurgii*.

[7] Wente M. N., Veit J. A., Bassi C. (2007). Postpancreatectomy hemorrhage (PPH)-an International Study Group of Pancreatic Surgery (ISGPS) definition. *Surgery*.

[8] Lee H. G., Heo J. S., Choi S. H., Choi D. W. (2010). Management of bleeding from pseudoaneurysms following pancreaticoduodenectomy. *World Journal of Gastroenterology*.

[9] Shimizu Y., Yasui K., Fuwa N., Arai Y., Yamao K. (2005). Late complication in patients undergoing pancreatic resection with intraoperative radiation therapy: gastrointestinal bleeding with occlusion of the portal system. *Journal of Gastroenterology and Hepatology*.

[10] de Castro S. M. M., Busch O. R. C., Gouma D. J. (2004). Management of bleeding and leakage after pancreatic surgery. *Best Practice & Research. Clinical Gastroenterology*.

[11] Grützmann R., Rückert F., Hippe-Davies N., Distler M., Saeger H. D. (2012). Evaluation of the International Study Group of Pancreatic Surgery definition of post-pancreatectomy hemorrhage in a high-volume center. *Surgery*.

[12] Yekebas E. F., Wolfram L., Cataldegirmen G. (2007). Postpancreatectomy hemorrhage: diagnosis and treatment - an analysis in 1669 consecutive pancreatic resections. *Annals of Surgery*.

[13] Asai K., Zaydfudim V., Truty M. (2015). Management of a delayed post-pancreatoduodenectomy haemorrhage using endovascular techniques. *HPB*.

[14] Zhang J., Zhu X., Chen H. (2011). Management of delayed post-pancreaticoduodenectomy arterial bleeding: interventional radiological treatment first. *Pancreatology*.

[15] Zhou T. Y., Sun J. H., Zhang Y. L. (2017). Post-pancreaticoduodenectomy hemorrhage: DSA diagnosis and endovascular treatment. *Oncotarget*.

[16] Gaudon C., Soussan J., Louis G., Moutardier V., Gregoire E., Vidal V. (2016). Late postpancreatectomy hemorrhage: predictive factors of morbidity and mortality after percutaneous endovascular treatment. *Diagnostic and Interventional Imaging*.

[17] Kasumova G. G., Eskander M. F., Kent T. S. (2016). Hemorrhage after pancreaticoduodenectomy: does timing matter?. *HPB*.

[18] Limongelli P. (2008). Management of delayed postoperative hemorrhage after pancreaticoduodenectomy. *Archives of Surgery*.

[19] Asari S., Matsumoto I., Toyama H. (2016). Recommendation of treatment strategy for postpancreatectomy hemorrhage: lessons from a single-center experience in 35 patients. *Pancreatology*.

[20] Suzumura K., Kuroda N., Kosaka H., Iimuro Y., Hirano T., Fujimoto J. (2014). Delayed arterial hemorrhage after pancreaticoduodenectomy. *International Surgery*.

[21] Biondetti P., Fumarola E. M., Ierardi A. M., Carrafiello G. (2019). Bleeding complications after pancreatic surgery: interventional radiology management. *Gland Surgery*.

[22] Iswanto S., Nussbaum M. L. (2014). Hepatic artery pseudoaneurysm after surgical treatment for pancreatic cancer: minimally invasive angiographic techniques as the preferred treatment. *North American Journal of Medical Sciences*.

[23] Miura F., Asano T., Amano H. (2009). Management of postoperative arterial hemorrhage after pancreato-biliary surgery according to the site of bleeding: re-laparotomy or interventional radiology. *Journal of Hepato-Biliary-Pancreatic Surgery*.

[24] Okuno A., Miyazaki M., Ito H. (2001). Nonsurgical management of ruptured pseudoaneurysm in patients with hepatobiliary pancreatic diseases. *The American Journal of Gastroenterology*.

[25] Roulin D., Cerantola Y., Demartines N., Schäfer M. (2011). Systematic review of delayed postoperative hemorrhage after pancreatic resection. *Journal of Gastrointestinal Surgery*.

[26] Rumstadt B., Schwab M., Korth P., Samman M., Trede M. (1998). Hemorrhage after pancreatoduodenectomy. *Annals of Surgery*.

[27] Ching K. C., Santos E., McCluskey K. M. (2016). Covered stents and coil embolization for treatment of postpancreatectomy arterial hemorrhage. *Journal of Vascular and Interventional Radiology*.

[28] Hassold N., Wolfschmidt F., Dierks A., Klein I., Bley T., Kickuth R. (2016). Effectiveness and outcome of endovascular therapy for late-onset postpancreatectomy hemorrhage using covered stents and embolization. *Journal of Vascular Surgery*.

